# Condom use among young women who sell sex in Zimbabwe: a prevention cascade analysis to identify gaps in HIV prevention programming

**DOI:** 10.1002/jia2.25512

**Published:** 2020-06-30

**Authors:** Sungai T Chabata, Bernadette Hensen, Tarisai Chiyaka, Phillis Mushati, Joanna Busza, Sian Floyd, Isolde Birdthistle, James R Hargreaves, Frances M Cowan

**Affiliations:** ^1^ Centre for Sexual Health and HIV/AIDS Research (CeSHHAR) Zimbabwe Harare Zimbabwe; ^2^ Faculty of Infectious and Tropical Diseases London School of Hygiene and Tropical Medicine London United Kingdom; ^3^ Faculty of Public Health and Policy London School of Hygiene and Tropical Medicine London United Kingdom; ^4^ Faculty of Epidemiology and Population Health London School of Hygiene and Tropical Medicine London United Kingdom; ^5^ Faculty of Clinical Sciences and International Public Health Liverpool School of Tropical Medicine Liverpool United Kingdom

**Keywords:** HIV prevention cascade, condom cascade, female sex worker, young women who sell sex, HIV prevention, Zimbabwe, sub‐Saharan Africa

## Abstract

**Introduction:**

Adolescent girls and young women (AGYW), including those who sell sex in sub‐Saharan Africa, are especially vulnerable to HIV. Reaching them with effective prevention is a programmatic priority. The HIV prevention cascade can be used to track intervention coverage, and identify gaps and opportunities for programme strengthening. The aim of this study was to characterise gaps in condom use and identify reasons underlying these gaps among young women who sell sex (YWSS) in Zimbabwe using data from enrolment into an impact evaluation of the DREAMS programme. DREAMS provided a package of biomedical, social and economic interventions to AGYW aged 10 to 24 with the aim of reducing HIV incidence.

**Methods:**

In 2017, we recruited YWSS aged 18 to 24 using respondent‐driven sampling in six sites across Zimbabwe. We measured knowledge about efficacy of, access to, and effective (consistent) use of condoms with the most recent three sexual partners, separately by whether YWSS self‐identified as female sex workers (FSW) or not. Among YWSS without knowledge about efficacy of, not having access to, and not effectively using condoms, we described the potential reasons underlying the gaps in the condom cascade. To identify socio‐demographic characteristics associated with effective condom use, we used logistic regression modelling. All analyses were RDS‐II weighted and restricted to YWSS testing HIV‐negative at enrolment.

**Results:**

We enrolled 2431 YWSS. Among 1842 (76%) YWSS testing HIV‐negative, 66% (n = 1221) self‐identified as FSW. 89% of HIV‐negative YWSS demonstrated knowledge about efficacy of condoms, 80% reported access to condoms and 58% reported using condoms consistently with the three most recent sexual partners. Knowledge about efficacy of and effective use of condoms was similar regardless of whether or not YWSS self‐identified as FSW, but YWSS self‐identifying as FSW reported better access to condoms compared to those who did not (87% vs 68%; age‐ and site‐adjusted (adjOR) = 2.69; 95% CI: 2.01 to 3.60; *p* < 0.001). Women who reported experiencing sexual violence in the past year and common mental disorder in the past week were less likely to use condoms consistently (43% vs. 60%; adjOR = 0.49; 95% CI: 0.35 to 0.68; *p* < 0.001) and (51% vs. 61%; adjOR = 0.76; 95% CI: 0.60 to 0.97; *p* = 0.029), respectively.

**Conclusions:**

Despite high knowledge about efficacy of and access to condoms, there remain large gaps in self‐reported consistent condom use among YWSS. Addressing the structural determinants of YWSS’ inconsistent condom use, including violence, could reduce this gap. YWSS who do not self‐identify as FSW have less access to condoms and may require additional programmatic intervention.

## 1 INTRODUCTION

Although HIV incidence is decreasing in sub‐Saharan Africa, it remains high among adolescent girls and young women (AGYW) aged 15 to 24 [1]. Among AGYW, young women who sell sex (YWSS) are at particularly high HIV risk [2], less likely to access health services, more likely to experience violence from sexual partners, and less likely to use condoms consistently due to a lower ability to negotiate condom use [3]. Reducing HIV incidence among AGYW requires increased coverage of efficacious prevention methods, including condoms and oral pre‐exposure prophylaxis (PrEP)[4, 5, 6].

The DREAMS Partnership was launched in ten African countries, including Zimbabwe, aiming to reduce HIV risk among AGYW through the delivery of comprehensive combination HIV prevention programming [7, 8]. In Zimbabwe, where YWSS, including young FSW, were among the target population, the core package included condom promotion and provision, and an offer of oral PrEP, supported by a range of behavioural and structural interventions [9].

HIV prevention cascades can highlight gaps in motivation for, access to and effective use of prevention tools [10, 11]. Criticisms of the framework emphasise that HIV prevention is non‐linear and complex, there are several prevention options, the prevention needs of individuals differ and change over time, and that measuring complex domains such as motivation, is challenging and not conducive to simplified models [12]. We operationalised a prevention cascade analysis for YWSS in Zimbabwe using data from enrolment into an impact evaluation of DREAMS. Overall, our goal was to explore whether application of prevention cascades at enrolment could be used to inform prevention programming by identifying priority interventions [13].

## 2 METHODS

### 2.1 Study setting and population

Between April and July 2017, we used respondent‐driven sampling (RDS) in six sites across Zimbabwe to identify and recruit YWSS to a cohort study to evaluate the impact of DREAMS on HIV incidence [14, 15]. Women were recruited from two large cities where DREAMS was implemented and a comparison group of women were recruited from four small towns selected based on their similarity with the DREAMS sites. In all six sites the national HIV prevention and treatment programme for FSW, “Sisters with a Voice”, provides support and services to FSW, including HIV testing, community mobilisation, and condoms.

Women were eligible to participate if they were 18 to 24 years of age and explicitly exchanged sex for money, goods or services in the past month. Our aim was to recruit any young women engaged in selling sex, even if these women did not see themselves as FSW. Based on the sample size required for the impact evaluation [14], we aimed to recruit ~600 YWSS in each large city, and ~300 in each small urban site.

### 2.2 Data collection

Data collection methods have been described elsewhere [14]. As reported [16], we conducted geographic and social mapping at each site to identify 6‐10 ‘seed’ participants, women who were purposefully selected to be representative of the social typology of YWSS, which is mainly street based, and geographic location of selling sex. Each ‘seed’ was interviewed and given two recruitment coupons to pass on to YWSS in her social network [14]. When YWSS receiving a coupon attended the survey site, they were given two coupons to pass on to two YWSS they knew, who sold sex in that location and who had not previously been recruited to the survey. Each participant was given an incentive of US$3 for participating in the survey, and an additional US$2 for each YWSS recruited. In all six sites, a maximum of six iterations of recruitment were performed [14].

YWSS consenting to participate completed a questionnaire on socio‐demographics, sexual behaviours, including a partner loop that asked about condom use at last sex and condom‐less sex in the past month with three most recent sexual partners, self‐identification as FSW, and uptake of HIV services, including testing. Participants were offered rapid HIV testing services according to national HIV testing guidelines and were told the result of their HIV test.

### 2.3 Measures

We operationalised the HIV prevention cascade measuring three core steps: knowledge about condom efficacy, access to and effective use of condoms [10, 11]. We used knowledge about condom efficacy as a proxy for motivation to use condoms because we had insufficient data to measure motivation to align the condom cascade with the Schaefer et al framework [10]. We hypothesised that cascades may differ by whether or not women self‐identified as FSW and constructed cascades for women who did and did not identify as such. All measures were self‐reported.

Knowledge about efficacy of condoms was defined as agreeing that using condoms every time you have sex can prevent an HIV negative person from acquiring HIV infection. Access to condoms was defined as reporting that condoms are always available at places where women choose to obtain them. Effective use of condoms was defined as having used condoms during all sexual acts with three most recent partners in the past month. Three recent partners were a smaller proportion of the average number of partners reported by YWSS self‐identified as FSW (20%) compared to those not self‐identified as FSW (60%). Effective condom use was a derived variable that combined data from two variables: condom use at last sex and condom‐less sex in the past month, with three most recent partners. The variable was coded 1 if women reported any condom‐less sex with any partner, and coded 0 only if they reported no condom‐less sex at last sex and in the last month with all three partners.

After constructing the cascade, we described perceived norms and perceptions about condom use among women without knowledge of condom efficacy, hypothesising that these might help better understand the gaps in their knowledge [13]. We described perceived use of condoms with (1) regular partners and (2) casual partners/clients by other young women, (3) perceived importance of using condoms with all sexual partners, and (4) whether using a condom every time they have sex is a good thing to do. Among women defined as not having access to condoms, we described whether women reported that: (1) it is easy for women like themselves to access free condoms, (2) it is expensive to travel to places where they get condoms, and (3) they are always able to get condoms for free at the places where they get condoms. Among women defined as not using condoms effectively, we described whether YWSS reported an ability to use condoms correctly, to negotiate condom use with any sexual partner, and confidence in their ability to ask a new sexual partner to use a condom. These measures related to self‐efficacy and skills were based on those developed and used in other settings in sub‐Saharan Africa [17, 18, 19, 20].

In a risk factor analysis, we included variables known to be associated with effective condom use in the literature [21] and that could be amenable to identifying women at risk of not using condoms and strategies to improve condom use, including: age, educational attainment, marital status, self‐identification as FSW, whether women ever experienced physical and sexual violence from a sexual partner or police, women’s relationship with other YWSS, number of close female friends, consumption of more than six alcoholic drinks in one night during last 12 months, and symptoms of common mental health disorders (CMD). Risk of CMD was assessed using the locally validated Shona Symptom Questionnaire (SSQ‐14) [22], a set of fourteen questions about symptoms of depression and anxiety in the previous one week (cut off for risk of CMD is ≥ 9/14) [22, 23, 24].

### 2.4 Statistical analysis

All analyses were restricted to women who tested HIV negative on the rapid HIV test offered during the survey. Data were RDS‐II weighted, with women’s responses weighted by the inverse of the reported number of YWSS that they knew i.e. the number of other women that she could have recruited to the survey [25], based on well established rationale for RDS‐II weighting [26, 27]. We pooled data from the six survey sites and normalised the RDS‐II weights by site. Participant socio‐demographic and sexual behaviour characteristics, as well as variables related to the cascade were described, and stratified by self‐identification as FSW.

We constructed the condom cascade for all YWSS as well as by whether or not they self‐identified as FSW, and compared each step by self‐identification as FSW adjusting for age at the time of the survey and site of recruitment.

Subsequently, we used logistic regression to identify socio‐demographic and sexual behaviour characteristics associated with effective condom use. For logistic regression analyses, we dropped seed participants and included a fixed term for site. Factors associated with effective condom use at *p* ≤ 0.10‐level in univariable analysis were included in the multivariable regression model, adjusting for all factors associated with effective condom use in the univariable analysis. We also explored whether the associations between the variables of interest and effective condom use were modified by whether or not women self‐identified as FSW. Evidence of effect modification in unadjusted analyses (*p* ≤ 0.10) resulted in further exploration in adjusted analyses.

Finally, we described potential reasons underlying gaps in the condom cascade among women defined as not having knowledge about condom efficacy, not having access to condoms, and not using condoms effectively. This analysis was also stratified by whether women self‐identified as FSW or not. Analyses were conducted using STATA version 14.2.

### 2.5 Ethics

The DREAMS impact evaluation was reviewed and approved by the Medical Research Council of Zimbabwe (Ref MRCZ/A/2085) and the London School of Hygiene and Tropical Medicine (Ref 11835). All participants were given information about the study and asked for written informed consent for participation.

## 3 RESULTS

### 3.1 Characteristics of women recruited to the study

We recruited 2431 YWSS, 1204 in two large cities and 1227 in the four small towns, 1842 (76%) tested HIV negative. The majority of HIV negative YWSS were aged 20 to 24 years (58%), had some but incomplete secondary school education, were never married, and reported having insufficient food in the past month (Table 1). Sixty‐six percent (66%) self‐identified as FSW. YWSS self‐identifying as FSW were older, more likely to be divorced/separated, more likely to consume alcohol and to report good relationships with other YWSS (Table 1). YWSS identifying as FSW reported having more sexual partners and sex work clients in the past month compared to YWSS not identifying as FSW. YWSS self‐identifying as FSW were also more likely to be at risk of CMD within the last week (37% vs. 27%), to have experienced physical and sexual violence from a sexual partner, and violence from police (6% vs. 2%) compared to non‐identifying YWSS.

**Table 1:** Socio‐demographic and sexual behavioural characteristics of YWSS testing HIV negative at enrolment into an RDS survey, by self‐identification as FSW, RDS‐II weighted (N = 1842)

	YWSS not identifying as FSW (N = 621)		YWSS identifying as FSW (N = 1221)		All YWSS (N = 1842)^a^		Comparison *p*‐value^c^
	n	%	n	%	n	%	
Age							<0.001
18 to 19	316	52.4	409	35.4	725	41.6	
20 to 24	305	47.6	812	64.6	1117	58.4	
Highest level of education							<0.001
None/ incomplete primary	18	2.7	87	7.7	105	5.9	
Complete primary	44	7.6	108	9.5	152	8.8	
Incomplete secondary	518	82.9	990	80.0	1508	81.1	
Complete secondary or higher	41	6.7	36	2.9	77	4.2	
Marital status							<0.001
Never been married	465	75.2	685	56.9	1150	63.6	
Married/ living together as if married	21	3.4	16	1.7	37	2.3	
Divorced/ separated	135	21.4	512	40.8	647	33.9	
Widowed	0	0.0	8	0.6	8	0.4	
Insufficient food in past month							0.002
No	308	50.5	517	41.7	825	44.9	
Yes	313	49.5	704	58.3	1017	55.1	
Age at start of selling sex							0.001
≤15	60	9.1	142	11.8	202	10.8	
16 to 17	246	41.5	392	31.7	638	35.3	
18 to 24	311	49.4	687	56.5	998	53.9	
Duration since first started selling sex (years)							<0.001
0 to 2	403	67.8	633	52.5	1036	58.0	
3 to 4	162	25.0	372	30.9	534	28.8	
≥5	52	7.2	216	16.7	268	13.2	
Number of sexual partners in the past month							<0.001
0 to 4	474	80.0	432	37.3	906	52.8	
5 to 9	82	10.9	303	25.9	385	20.5	
≥10	64	9.1	481	36.8	545	26.7	
Number of clients in the past month							<0.001
0 to 4	501	84.1	485	42.1	986	57.4	
5 to 9	62	7.8	276	23.6	338	17.9	
≥10	57	8.1	454	34.3	511	24.8	
Relationship with other YWSS							<0.001
Good	385	63.6	869	71.6	1254	68.7	
Neither good nor bad	125	21.2	229	18.6	353	19.5	
Bad	60	10.3	97	8.2	157	9.0	
No relationship	48	4.9	26	1.5	74	2.7	
No. of close female friends							0.231
0	127	22.0	219	19.7	346	20.5	
1	356	56.8	667	55.0	1023	55.7	
≥2	138	21.2	335	25.3	473	23.8	
Alcohol consumption in the past 12 months							<0.001
Never	354	60.1	480	41.7	834	48.4	
Once a month or less	103	17.6	159	13.4	262	15.0	
2 to 4 times per month	67	9.7	183	14.5	250	12.7	
2 to 3 times per week	60	8.3	212	16.5	272	13.5	
4 or more times per week	37	4.3	187	13.9	224	10.4	
Had more than six alcoholic drinks in one night during last 12 months							<0.001
Have not had alcohol in last 12 months	354	60.1	480	41.7	834	48.4	
Drank alcohol but no occasions of more than six drinks	160	25.3	386	32.2	546	29.7	
Yes, at least one occasion	107	14.6	355	26.1	462	21.9	
Risk of CMD^b^							<0.001
No	443	72.7	768	62.9	1211	66.4	
Yes	178	27.3	453	37.1	631	33.6	
Ever experienced physical violence from sexual partner							<0.001
No	437	70.5	707	60.3	1144	64.0	
Yes	184	29.5	514	39.7	698	36.0	
Experienced sexual violence from a sexual partner in the past 12 months							0.010
No	541	88.3	1005	82.9	1546	84.9	
Yes	79	11.7	216	17.1	295	15.1	
Experienced any form of violence from police in the past 12 months							<0.001
No	606	98.3	1150	94.4	1756	95.8	
Yes	13	1.7	69	5.6	82	4.2	

*p*‐value is from Wald test. YWSS, young women who sell sex; FSW, female sex worker; RDS, respondent‐driven sampling.

a 17 women missing data on whether or not they self‐identify as FSW.

b Cut‐off of ≥9.

c Adjusted for age and site. CMD, common mental health disorders.

### 3.2 The condom cascade

**Figure 1:**
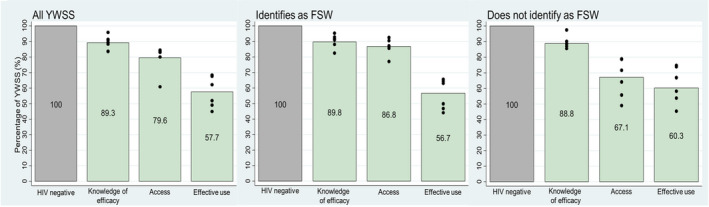
Condom cascades among YWSS overall, YWSS self‐identifying as FSW and YWSS not self‐identifying as FSW. Black spots indicate site‐specific estimates. YWSS, young women who sell sex; FSW, female sex worker; FSW, female sex workers.

Overall, 89% of YWSS agreed that using condoms every time during sex can prevent an HIV negative person from acquiring HIV infection (efficacy knowledge), 80% reported that condoms were always available at places from which they chose to obtain them (access), and 58% reported having used condoms during all sexual acts with three most recent partners in the past month (effective use) (Figure 1).

A higher proportion of YWSS self‐identifying as FSW reported access to condoms compared to YWSS not identifying as FSW (87% vs. 67%; age‐ and site‐adjusted OR = 2.69; 95% CI: 2.01 to 3.60; *p* < 0.001). Knowledge about efficacy of condoms and effective use of condoms were similar between both groups of YWSS (90% vs. 89%; OR = 1.00; 95% CI: 0.68 to 1.47; *p* = 0.997), and (57% vs. 60%; OR = 0.94; 95% CI: 0.61 to 1.28; *p* = 0.123), respectively.

Adjusting for factors associated with effective condom use in univariable analysis, there was evidence that effective condom use was lower among YWSS at risk of CMD (51% vs. not at risk 61%; adjOR = 0.76; 95% CI: 0.60 to 0.97; *p* = 0.029) and women who ever experienced physical violence from a sexual partner (51% vs. women not experiencing physical violence 62%; adjOR = 0.74; 95% CI: 0.58 to 0.96; *p* = 0.021) (Table 2). There was strong evidence that effective condom use was lower among women who had experienced sexual violence in past 12 months compared to women reporting no sexual violence (43% vs. 60%; adjOR = 0.49; 95% CI: 0.35 to 0.67; *p* < 0.001). YWSS who reported neither a good nor bad relationship with other YWSS were less likely to use condoms effectively compared to YWSS with good relationship with other YWSS (50% vs. 60%; adjOR = 0.49; 95% CI: 0.35 to 0.68; *p* = 0.030). There was little evidence of an association with other factors explored and no statistical evidence for any effect modification by self‐identification as FSW.

**Table 2:** Factors associated with effective condom use among YWSS testing HIV negative at enrolment into an RDS survey, RDS‐II weighted (N = 1810)

Characteristic	N (%)	# of YWSS who had effective condom use in the past month (N = 1058) n (%)	Crude OR (95% CI)	*p*‐value	Adjusted OR^a^ (95% CI)	*p*‐value
Age at enrolment				0.039		0.965
18 to 19	712 (41.5)	432 (59.9)	1		1	
20 to 24	1098 (58.5)	626 (56.1)	0.78 (0.62 to 0.99)		0.99 (0.76 to 1.30)	
Highest level of education				0.584		
Primary or less	253 (14.6)	141 (55.4)	1			
Incomplete secondary	776 (42.9)	476 (58.5)	1.20 (0.85 to 1.69)			
Complete secondary or higher	781 (42.5)	441 (57.6)	1.16 (0.82 to 1.65)			
Marital status				0.003		0.062
Never married	1130 (63.7)	667 (59.4)	1		1	
Ever married	680 (36.3)	391 (54.7)	0.69 (0.54 to 0.89)		0.77 (0.59 to 1.01)	
Insufficient food in past month				0.506		
No	807 (44.8)	466 (55.5)	1			
Yes	1003 (55.2)	592 (59.4)	1.08 (0.86 to 1.36)			
Self‐identified as FSW				0.023		0.386
No	598 (35.8)	356 (60.3)	1		1	
Yes	1195 (64.2)	696 (56.7)	0.75 (0.59 to 0.96)		0.89 (0.69 to 1.16)	
Age at start of selling sex				0.166		
≤15	196 (10.8)	103 (50.2)	1			
16 to 17	637 (35.7)	363 (54.9)	1.15 (0.79 to 1.69)			
18 to 24	974 (53.5)	590 (60.9)	1.36 (0.95 to 1.96)			
Duration since first started selling sex (years)				0.003		0.054
0 to 2	1015 (57.7)	631 (61.1)	1		1	
3 to 4	530 (29.0)	293 (54.9)	0.81 (0.63 to 1.04)		0.86 (0.66 to 1.12)	
≥5	262 (13.3)	132 (48.3)	0.58 (0.42 to 0.80)		0.64 (0.45 to 0.91)	
Relationship with other YWSS				0.025		0.030
Good	1238 (69.1)	746 (60.2)	1		1	
Neither good nor bad	345 (19.5)	182 (49.7)	0.67 (0.51 to 0.90)		0.67 (0.50 to 0.90)	
Bad or no relationship	223 (11.4)	127 (56.1)	0.88 (0.62 to 1.24)		0.88 (0.63 to 1.25)	
No. of close female friends				0.578		
0	342 (20.7)	198 (57.8)	1			
1	1008 (55.6)	584 (57.1)	0.99 (0.75 to 1.34)			
≥2	460 (23.7)	276 (58.8)	1.15 (0.82 to 1.62)			
Had more than six alcoholic drinks in one night during last 12 months				0.032		0.450
Have not had alcohol in last 12 months	816 (48.1)	506 (61.7)	1		1	
Drank alcohol but no occasions of more than six drinks	542 (29.9)	319 (55.4)	0.74 (0.56 to 0.96)		0.85 (0.64 to 1.11)	
Yes, at least one occasion	452 (22.0)	233 (51.9)	0.72 (0.54 to 0.97)		0.99 (0.72 to 1.35)	
Risk of CMD in the past week				<0.001		0.029
No	1182 (66.1)	737 (61.0)	1		1	
Yes	628 (33.9)	321 (51.1)	0.66 (0.52 to 0.83)		0.76 (0.60 to 0.97)	
Ever experienced physical violence from sexual partner				<0.001		0.021
No	1122 (64.1)	697 (61.5)	1		1	
Yes	688 (35.9)	361 (50.8)	0.60 (0.48 to 0.76)		0.74 (0.58 to 0.96)	
Experienced sexual violence from a sexual partner in the past 12 months				<0.001		<0.001
No	1518 (84.7)	930 (60.3)	1		1	
Yes	291 (15.3)	127 (42.6)	0.43 (0.31 to 0.58)		0.49 (0.35 to 0.67)	
Experienced any form of violence from police in the past 12 months				0.003		0.239
No	1726 (95.9)	1016 (58.4)	1		1	
Yes	80 (4.1)	40 (40.4)	0.45 (0.27 to 0.77)		0.71 (0.41 to 1.25)	

*p*‐value is from Wald test. YWSS, young women who sell sex; FSW, female sex worker; RDS, respondent‐driven sampling; OR, odds ratio.

a Adjusted for all factors associated with effective condom use in crude analysis.

### 3.3 Potential reasons underlying lack of knowledge about efficacy of, access to and effective use of condoms

**Figure 2:**
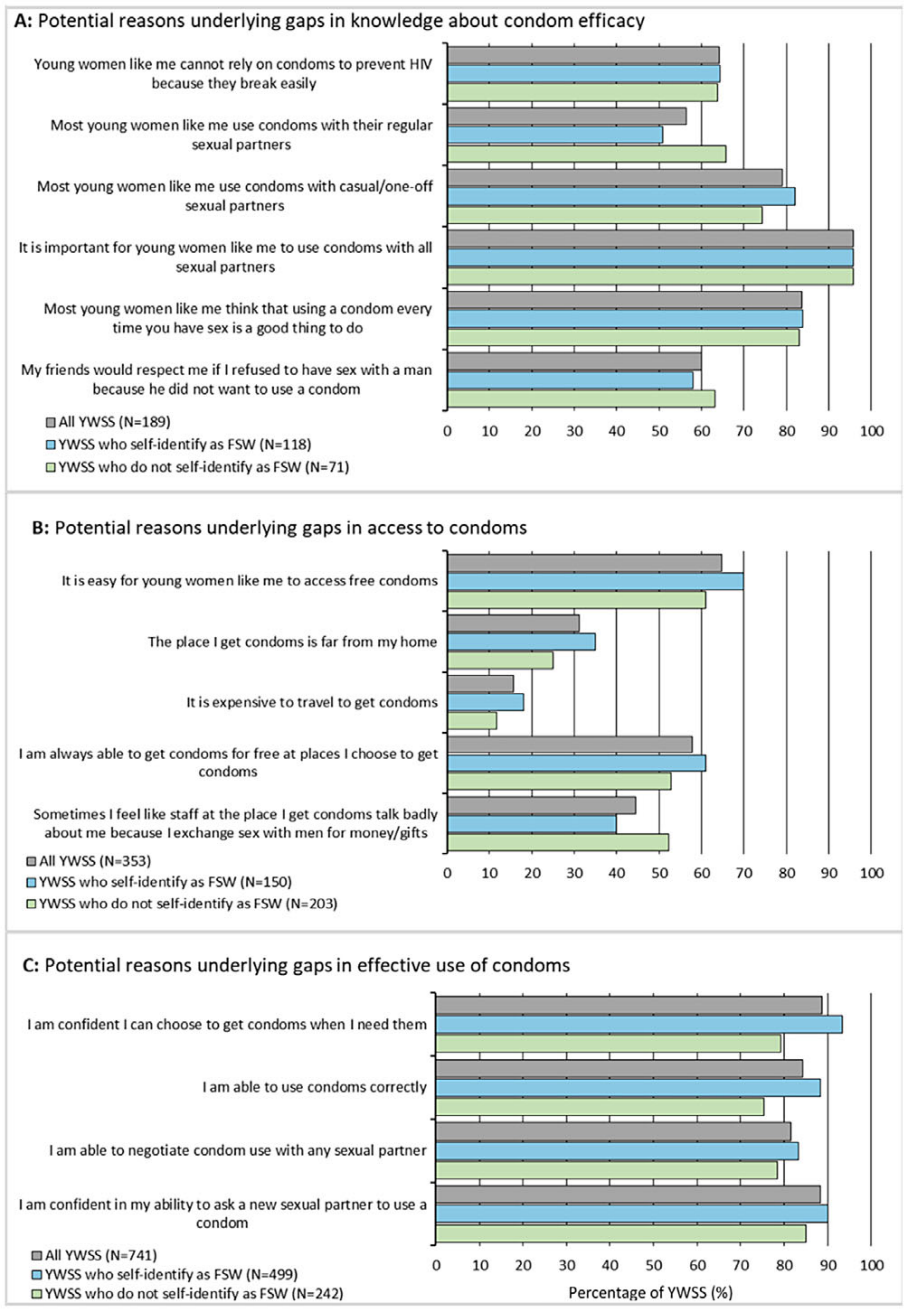
Potential reasons underlying gaps in knowledge about efficacy of, access to and effective use of condoms among YWSS without knowledge about efficacy of, not having access to, and not effectively using condoms, respectively. YWSS, young women who sell sex.

Among YWSS who did not perceive condoms to be effective at preventing HIV, the majority reported that other YWSS use condoms with their casual partners/clients (80%) and regular partners (58%). Most women perceived the use of condoms with all sexual partners as important (97%) and considered using condoms every sex act as a good thing to do (84%). A high proportion (68%) of women reported that they cannot rely on condoms because they break easily (Figure 2A). There was little evidence that these factors differed by self‐identification as FSW.

Among YWSS who lacked access to condoms, the majority (64%) reported that it is easy for young women like them to access free condoms and that they are always able to get condoms for free (58%). Access to free condoms was similar regardless of whether women self‐identified as FSW or not (70% vs 61% respectively; age‐ and site‐adjusted (adjOR)=1.43; 95% CI: 0.82 to 2.47; *p* = 0.204) (Figure 2B). Few women (31%) reported that places to get condoms are far from their homes or that it is expensive to travel to get condoms (16%), with no evidence for difference by how YWSS self‐identified. Forty‐percent (40%) reported that staff at places where they get condoms talk badly about them because they sell sex, with no evidence for a difference by self‐identification as FSW.

Among YWSS not using condoms effectively, YWSS self‐identifying as FSW were more likely to report that they were confident to get condoms when they needed them (92% vs. 79%; adjOR = 2.61; 95% CI: 1.41 to 4.84; *p* = 0.002) and were able to use condoms correctly (87% vs. 75%; adjOR = 1.95; 95% CI: 1.15 to 3.31; *p* = 0.013) compared to YWSS not identifying as FSW. Ability to negotiate condom use with any sexual partner and confidence in one’s ability to ask a new sexual partner to use a condom was high and similar between both groups of YWSS (Figure 2C).

## 4 DISCUSSION

We operationalised a condom cascade using data collected from HIV‐negative YWSS in six sites in Zimbabwe in 2017. Knowledge about the efficacy of condoms was high. However, reported effective use with their three most recent partners was low and access to condoms differed between YWSS who self‐identified as FSW and those who did not. YWSS perceived that their peers used condoms and considered condoms important. Yet condoms were not considered reliable, YWSS not identifying as FSW had less access to free condoms and lower condom use self‐efficacy. Effective condom use was lower among those experiencing violence and being at risk of CMD, suggesting that these factors affect women’s capability to use condoms.

Our analysis revealed gaps in access to condoms and in confidence in condom use, particularly among YWSS not identifying as FSW, who were younger and started selling sex more recently. These gaps point to the need for tailored programming depending on how YWSS self‐identify [21, 28]. Demand creation for condoms may have been neglected in recent years. Re‐enforcing the message that condoms are effective when used correctly, coupled with promotion of how to use condoms properly and that they should be used consistently, is critical. At the time of our study, PrEP was not widely available; few women had heard of PrEP. Currently, there is little literature on motivation to use PrEP and factors affecting ability to adhere to PrEP [29]. Prevention cascades may inform our thinking about how to support PrEP use and adherence, highlighting that alongside access and knowledge of PrEP efficacy, programmes need to consider self‐efficacy and confidence, and how violence and CMD are likely to affect longer‐term PrEP use. Further, our study suggests the need to promote PrEP as an option for women who feel they cannot negotiate condom use because they are at risk of violence, or who may struggle to negotiate condom use with partners who insist on condom‐less sex [30].

Self‐efficacy to use condoms is likely compromised by physical and sexual violence from sexual partners. HIV prevention programmes need to provide access to violence prevention services, including violence mitigation packages [31, 32]. Women participating in the survey reported high levels of violence, and our analysis of the final cascade step revealed that experiencing violence was associated with reported ineffective use of condoms with their three most recent partners. These findings are similar to other studies [33, 34], which have shown that intimate partner violence is associated with poorer condom use self‐efficacy and condom use [32, 35]. By measuring structural factors that may influence steps along a cascade, programmes would be better placed to understand factors influencing service use and to support YWSS to access a holistic package of services. Included in the DREAMS package of interventions was post‐violence care, school‐based HIV and violence prevention, social protection interventions and community mobilisation [8]. Greater investments in strategies to reduce violence experienced by YWSS in particular [3] alongside interventions targeting the male partners of YWSS are needed. Access to such interventions could be included in monitoring targets for DREAMS among key populations, alongside HIV testing, condoms and PrEP.

This study has several strengths. We recruited a large number YWSS from multiple sites using a similar RDS method, and included YWSS who do and do not self‐identify as FSW who are often missed by research and HIV prevention programmes [36]. Our RDS diagnostics (reported elsewhere [21, 37]) suggest that our sample is likely to be representative of the network of YWSS recruited. These analyses gave us a unique opportunity to operationalise a prevention cascade within a large group of HIV‐negative YWSS, recruited for an impact evaluation of DREAMS, among whom data on condom use is limited in sub‐Saharan Africa [38].

A limitation of the data we report is our reliance on self‐reports of subjects prone to social desirability bias. Approaches to strengthen valid reporting, including ACASI, are available but were not possible to use in these surveys. We were not able to include detailed questions about partner’s influence in determining condom use. However, understanding women’s agency in condom negotiation with individual partners may be better placed for qualitative inquiry, particularly among those women who reported experiencing violence and for whom violence was strongly associated with effective condom use [14, 15].

The cascades we present have limitations. In the absence of a standard measure for motivation [10], we used knowledge about the efficacy of condoms as the first step in our cascade. Knowledge that condoms can prevent HIV is necessary but not sufficient for motivation. We described norms and perceptions regarding condom use, which revealed that many women consider condoms unreliable. Our measure of access is also limited. Condoms always being available may not mean women have access if the places where condoms are available are not acceptable [10]. Discrimination by staff may deter YWSS from accessing condoms [39]. Among YWSS defined as lacking access to condoms, some reported that staff at the place they get condoms talk badly about them because they sell sex. Our measure of effective condom use may also over‐report consistent condom use. Women’s behaviours with their three most recent partners may not be reflective of behaviours with all partners. Also, YWSS could be having condom‐less sex due to pregnancy intentions, particularly with a non‐paying partner. We estimate that our approach focusing on the three most recent partners covered at least 50% of partners in the last month. As such, and despite limitations, our measure is similar to those reported in other studies [40]. The questions to quantify reasons for any gaps in knowledge about efficacy, access and effective use were related to domains considered of importance in the cascade presented by Schaefer et al [10] and Hargreaves et al [11], and combine behavioural theories [41]. There may, however, be reasons underlying gaps in access to condoms among YWSS who do not self‐identify as FSW and in effective use among all women that we failed to measure, including whether women disclosed their sex work to friends, family and partners.

A further limitation is that the prevention cascade is intended to be a simple and practical framework to strengthen prevention programming [10, 42]. Although the cascades we present are in themselves simple, the collection of data through RDS surveys is complex and such data is not routinely available to programmes. However, questions included in the survey or related to motivation and access could be collected by peer educators or through micro‐planning methods, with qualitative data collection methods used to complement quantitative findings. Data routinely collected by FSW programmes in Kenya and in Zimbabwe [43, 44] provided powerful information to strengthen programming, revealing gaps in reaching younger FSW [42]. Combining survey data, when available, with data collection by peer educators, including micro‐planning, could prove a powerful tool to identify gaps and strengthen programming for YWSS in Zimbabwe and other settings [42, 45].

## 5 CONCLUSIONS

We used the HIV prevention cascade to determine effective condom use among YWSS at high risk of HIV and identify programme gaps and possible strategies to increase condom use. This approach was very useful in identifying gaps but needs to be complemented by qualitative enquiry to better understand why gaps exist.

## COMPETING INTERESTS

No competing interests to declare.

## AUTHORS’ CONTRIBUTIONS

SC planned and conducted the analysis, and wrote the first draft; BH was involved in planning the analysis and contributed to writing; TC and PM led the data collection; JB provided critical review of the article, particularly the introduction; SF, IB, JH, and FC were involved in the conception of the study, and BH, FC and JH critically revised the article. All authors contributed to the writing and have read and approved the final version.
